# Differential synaptic mechanism underlying the neuronal modulation of prefrontal cortex, amygdala, and hippocampus in response to chronic postsurgical pain with or without cognitive deficits in rats

**DOI:** 10.3389/fnmol.2022.961995

**Published:** 2022-09-02

**Authors:** Zhen Li, Zhigang He, Zhixiao Li, Tianning Sun, Wencui Zhang, Hongbing Xiang

**Affiliations:** Department of Anesthesiology and Pain Medicine, Tongji Hospital of Tongji Medical College, Huazhong University of Science and Technology, Wuhan, China

**Keywords:** chronic postsurgical pain, cognitive function, excitatory postsynaptic currents (EPSCs), inhibitory postsynaptic synaptic currents (IPSCs), postsynaptic density

## Abstract

Chronic Postsurgical Pain (CPSP) is well recognized to impair cognition, particularly memory. Mounting evidence suggests anatomic and mechanistic overlap between pain and cognition on several levels. Interestingly, the drugs currently used for treating chronic pain, including opioids, gabapentin, and NMDAR (N-methyl-D-aspartate receptor) antagonists, are also known to impair cognition. So whether pain-related cognitive deficits have different synaptic mechanisms as those underlying pain remains to be elucidated. In this context, the synaptic transmission in the unsusceptible group (cognitively normal pain rats) was isolated from that in the susceptible group (cognitively compromised pain rats). It was revealed that nearly two-thirds of the CPSP rats suffered cognitive impairment. The whole-cell voltage-clamp recordings revealed that the neuronal excitability and synaptic transmission in the prefrontal cortex and amygdala neurons were enhanced in the unsusceptible group, while these parameters remained the same in the susceptible group. Moreover, the neuronal excitability and synaptic transmission in hippocampus neurons demonstrated the opposite trend. Correspondingly, the levels of synaptic transmission-related proteins demonstrated a tendency similar to that of the excitatory and inhibitory synaptic transmission. Furthermore, morphologically, the synapse ultrastructure varied in the postsynaptic density (PSD) between the CPSP rats with and without cognitive deficits. Together, these observations indicated that basal excitatory and inhibitory synaptic transmission changes were strikingly different between the CPSP rats with and without cognitive deficits.

## Introduction

Chronic postsurgical pain (CPSP) affects nearly 10–50% of patients after surgery (Kehlet et al., 2006), impacting their quality of life negatively. CPSP is reported to impair cognition, including learning and memory (Cardoso-Cruz et al., 2013; Suto et al., 2014; Kehlet, 2018), which could be on account of pain using up a significant proportion of the limited cognitive resources of patients (Attal et al., 2014; Phelps et al., 2021). Conversely, cognitive functions may also influence the pain experienced by the patients (Bushnell et al., 2013; Matthewson et al., 2019; Howlin and Rooney, 2020). However, despite the universal agreement on the clinical significance of pain-related cognitive deficits, the underlying mechanisms have not been elucidated so far.

Previous studies have proposed the attentional cost hypothesis, brain plasticity, and structural changes in the brain as the possible mechanisms (Moriarty and Finn, 2014; Phelps et al., 2021). Depression, anxiety, sleep disturbances, fatigue, age, and generalized diffuse pain states might also be involved, according to certain studies (Mazza et al., 2018). However, in all of these previous studies, the animals that suffered chronic pain were placed in a single group without considering the difference between cognitively compromised pain animals and cognitively normal pain animals. Moreover, the drugs currently used for treating chronic pain, including gabapentin, opioids, and NMDAR (N-methyl-D-aspartate receptor) antagonists, are also indicated to impair memory (Kamboj et al., 2005; Morgan et al., 2014; Shem et al., 2018; Phelps et al., 2021). Therefore, it is of great importance to comprehensively investigate whether the pain-related cognitive deficits have different underlying mechanisms as those underlying the pain symptom.

As in the case of clinical surgical procedures, skin/muscle incision and retraction (SMIR) also leads to mechanical hypersensitivity, which is maintained mainly by supraspinal rather than purely spinal dysfunction (Flatters, 2008; Ying et al., 2014; Fitzcharles et al., 2021). In addition, the CPSP arising due to SMIR is not driven by neuronal damage because of a lack of either demyelination or injury in the saphenous nerve, further indicating the involvement of relevant central nervous system-related mechanisms (Flatters, 2008; Ying et al., 2014). Research has revealed that CPSP is the consequence of either nerve injury-induced neuropathic pain or the continuing inflammation (Kehlet et al., 2006). In this context, the SMIR model could be particularly relevant for the inflammation-induced pain that arises as a consequence of the elevated excitability of neurons in the central nervous system (central sensitization) (Kehlet et al., 2006). Moreover, studies associating synaptic transmission to pain and cognition are emerging (McCarberg and Peppin, 2019; Xiong et al., 2020). Collectively, these findings suggest that alterations in synaptic transmission might be associated with pain-related cognitive impairments. Anatomically, the medial prefrontal cortex (mPFC), central amygdala (CeA), and hippocampus, which are central to the modulation of cognition, are also implicated in the regulation of pain (Moriarty and Finn, 2014; McCarberg and Peppin, 2019; Phelps et al., 2021). Therefore, in the present study, the difference between the cognitively compromised pain animals and cognitively normal pain animals in terms of basal synaptic transmission from the mPFC, CeA, and hippocampal CA1 neurons was investigated.

## Materials and methods

### Experimental animals

Sprague-Dawley (SD) rats (male, weighing 200 ± 10 g each) procured from the Animal Center of Tongji Hospital were used in the experiments. All animals were housed under standard conditions (temperature 22°C–24°C, 12-h light/12-h dark photocycle) with *ad libitum* access to food and water. The experimental protocols were approved by the Animal Care and Use Committee, Tongji Hospital. All experiments were conducted by strictly following the instructions provided in the Guide for the Care and Use of the Laboratory Animals, National Institute of Health.

### Behavior test

In order to assess whether the SMIR surgery evoked a significant mechanical hypersensitivity, mechanical sensitivity was measured. Subsequently, the open-field test was conducted to evaluate the locomotor activity (Liu et al., 2020). Finally, the Y-maze and novel object preference (NOP) tests were performed to estimate the degree of cognitive impairment induced by persistent pain hypersensitivity (Mathiasen and DiCamillo, 2010; Liu et al., 2020; Yuan et al., 2020). The cognitive behavior of each animal was monitored and analyzed simultaneously using an animal tracking system software (ANY-maze, Stoelting). After each trial, the test devices were cleaned to avoid interference due to odor. The results of the hierarchical clustering analysis in the Y-maze and the NOP tests revealed that the animals that suffered chronic pain could be classified into two groups: the unsusceptible group (comprising cognitively normal pain rats) and the susceptible group (comprising cognitively compromised pain rats).

### Assessment of mechanical sensitivity

Mechanical sensitivity was assessed based on the paw withdrawal threshold (PWT) test using grade-strength von Frey monofilaments (1.0–15 g) as described in a previous report (Li et al., 2016, 2017). Prior to the test, the rats were placed inside separate plastic chambers over mesh platforms and acclimated for over 30 min to achieve immobility. Subsequently, according to the up–down paradigm, beginning with a quantity of 1.0 g, filaments of sequentially increasing stiffness were applied vertically to the mid-plantar surface of the right hind paw of each rat until the filament bent. A positive response was defined as brisk withdrawal or licking the paw. A minimum of 5 min interval was maintained between adjacent tests.

### Open field test

After 1 h of acclimatization to the testing room, each rat was placed in the center of a black open-field chamber (size 100 × 100 × 40 cm), where it was allowed to move spontaneously for 5 min. The total distance traveled by the rat was recorded and used for measuring the rat’s locomotor activity (Liu et al., 2020).

### Novel object preference test

The NOP test was conducted to evaluate the non-spatial visual learning memory. The test was conducted within a black open field with two stages. At the training stage, after accommodation without objects, the rat was allowed for 5 min to freely explore the two exact things pasted on the central symmetrical positions of the field. Two hours later, the rat was placed in the same region again and allowed to free exploration for 5 min once again, this time with one of the objects being replaced by a novel, unfamiliar object (test session). The time spent exploring the familiar object (F), and the novel object (N) was recorded. The recognition index, reflecting the memory function, was calculated using the formula (N-F)/(N + F)*100% (Mathiasen and DiCamillo, 2010; Xiong et al., 2020).

### Y-maze test

The Y-maze test was conducted to evaluate the spatial orientation learning memory ability of the rats. The Y-maze comprised three identical arms that converged to an equal angle. The randomly named start arm (animal entry) and the other arm remained open always. Initially, the new arm was blocked, while the other two arms were available to the rat for free exploration for 5 min. After 2 h, all the arms were opened, allowing the rat free access to all three arms for 5 min. The entries into the new arm and the time spent in the new arm reflected the spatial recognition memory (learned behavior) (Liu et al., 2020).

### Skin/muscle incision and retraction model

The SMIR surgery of rats was performed as described in previous reports (Flatters, 2008; Liu et al., 2021). The rats were anesthetized using pentobarbital sodium (50 mg/kg, intraperitoneally) and placed on their backs, followed by shaving the right medial thigh region and sterilizing it using alcohol. A 1.5–2 cm incision (approximately 4 mm medial to the saphenous vein) was performed in the skin of the medial thigh to expose the muscle of the thigh. Next, a 1 cm incision (approximately 3 mm proximal to the saphenous nerve) was performed in the superficial muscle layer. Subsequently, the external muscle was parted via a blunt dissection to insert a retractor into the incision site. The skin and superficial muscle of the thigh were retracted by 2 cm to expose the fascia underlying the adductor muscles for 1 h. Sham rats underwent the same procedure without skin/muscle retraction. The main objective of the SMIR model was to mimic the clinical scenario, in which persistent postoperative pain was evoked by SMIR rather than by neuronal damage.

### Electrophysiology of brain slices

**FIGURE 1:**
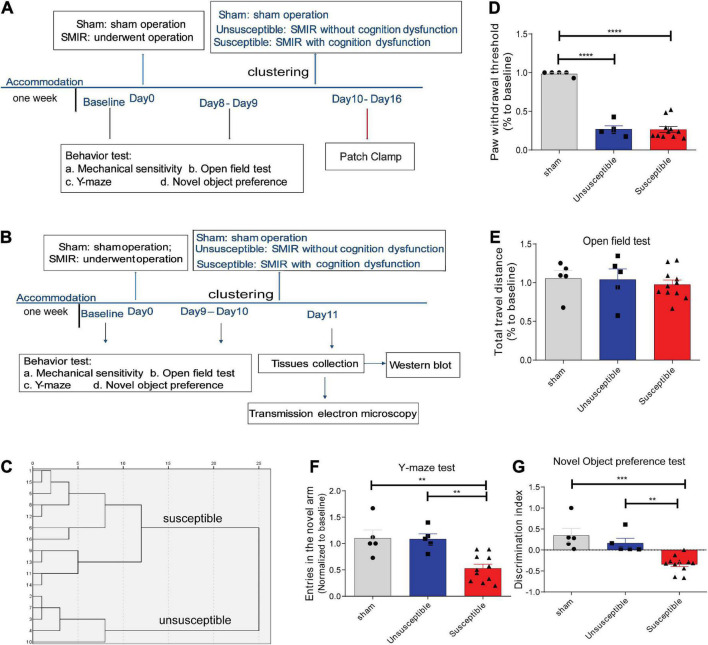
SMIR-induced significant mechanical hypersensitivity and cognitive impairment in rats. **(A,B)** Schematic illustration of the design of the complete experiment. **(C)** Dendrogram of the hierarchical clustering analysis. The rats who underwent the SMIR surgery were divided into the susceptible group (CPSP rats with cognitive dysfunction, *n* = 11) and the unsusceptible group (CPSP rats without cognitive dysfunction, *n* = 5) according to the hierarchical clustering analysis of the data from the Y-maze test and the novel object preference test. **(D–G)** Behavioral test after SMIR surgery, including **(D)** mechanical sensitivity (*F* = 90.07, *p* < 0.0001), **(E)** the open field test (*F* = 0.28, *p* = 0.76), **(F)** the Y-maze (*F* = 11.40, *p* = 0.0006), **(G)** the novel object preference test (*F* = 14.21, *p* = 0.0002). Results are expressed as mean ± SEM; Tukey’s *post-hoc* tests; **p < 0.01, ***p < 0.001, ****p < 0.0001.

As described previously (Li et al., 2016, 2017), whole-cell recordings from the anterior cingulate cortex (the dorsal component of mPFC), hippocampal CA1, and CeA neurons were obtained and analyzed to reveal the alterations in synaptic transmission. After the behavioral tests (Figure 1A), rats in each group were anesthetized using 2% sodium pentobarbital (30 mg/kg, intraperitoneal injection), and their brain tissues were dissected and placed into a cutting solution at 4°C. The dissection solution comprised the following (in mM): glucose, 10; sucrose, 213; KCl, 3; NaHCO_3_, 26; NaH_2_PO_4_, 1.2; CaCl_2_, 0.5; MgCl_2_, 5. After a recovery period of at least 1 h at room temperature in oxygenated artificial cerebrospinal fluid (ACSF), an individual slice was transferred to a submersion-recording chamber that was being continuously perfused with ACSF at a flow rate of 2 mL/min. The ACSF comprised the following (in mM): NaCl, 125; NaHCO_3_, 26; KCl, 5; NaH_2_PO_4_, 1.2; CaCl_2_, 2.6; MgCl_2_, 1.3; glucose, 10. Only one neuron was recorded from each slice. In one animal, two neurons were recorded from mPFC, CeA, and hippocampal CA1 each.

The whole-cell recordings from visually identified ACC, CeA, and hippocampal CA1 excitatory pyramidal neurons were obtained using an Axonpatch 700B amplifier (Molecular Devices, United States). The recordings were digitized using a Digidata 1500B digital converter (Molecular Devices, United States). The spontaneous and miniature excitatory postsynaptic currents (sEPSCs/mEPSCs) and the spontaneous and miniature inhibitory postsynaptic currents (sIPSCs/mIPSCs) were recorded at –70 mV. The WPI recording pipettes (4–8 MΩ, United States) were filled with an intracellular solution comprising the following (in mM): potassium-gluconate, 122; NaCl, 5; MgCl_2_, 2; CaCl_2_, 0.3; HEPES, 10; EGTA, 5; Mg-ATP, 4; Na_3_-GTP, 0.3 (pH 7.30–7.35 and 300 mOsm/kg). The recording was carried out using microelectrodes filled with K + gluconate (for action potentials and sEPSCs/mEPSCs recordings) or KCl (for sIPSCs/mIPSCs determinations) (Sceniak et al., 2016). The KCl solution consisted of (in mM): 100 KCl, 40 HEPES, 0.2 EGTA, 5 MgCl_2_, 2 Mg-ATP, 0.3 Na_3_-GTP, 10 phosphocreatine. The mEPSCs were recorded with picrotoxin (100 μM) and tetrodotoxin (TTX) (1 μM) added to the ACSF, and the mIPSCs were pharmacologically isolated using CNQX (10 μM), AP-5 (100 μM), and TTX (1 μM). The sEPSCs/sIPSCs were recorded without TTX added to the ACSF. The excitatory pyramidal neurons and the inhibitory interneurons in different regions were identified by accommodating responses to a sustained depolarizing intracellular current stimulation. Regular spiking neurons with a firing rate of < 5 Hz were believed to correspond to the excitatory pyramidal neurons, while the fast-spiking neurons with a firing rate of > 10 Hz and shorter duration waveforms were believed to correspond to the inhibitory interneurons (Laviolette and Grace, 2006; Koyanagi et al., 2021). We only recorded the excitatory neurons. The number of action potentials (APs) evoked upon the intracellular current injections of increasing magnitude (from 0 to 300 pA, with steps of 50 pA; duration 500 ms) was counted to determine the passive membrane property and the single AP characteristics without using any synaptic blocker. High seal (GΩ) and low series (30 MΩ) resistances were monitored throughout the experiment (using the membrane test function) to ensure a high-quality recording. A fixed length of traces (5 min) was analyzed to obtain the frequency and amplitude of sEPSCs/sIPSCs or mEPSCs/mIPSCs with pCLAMP10.7.

### Western blotting

Immediately after the rats were euthanized (Figure 1B), their mPFC, CeA, and hippocampal CA1 were retrieved immediately through dissection and stored at –80°C until analysis (Li et al., 2016, 2017). The tissues were homogenized and lysed using a radio-immuno-precipitation assay buffer (AR0102, Boster) and then subjected to the measurement of protein concentration using the bicinchoninic acid kit (AR1189, Boster). The lysates (20 μg each) were separated on 10% SDS-PAGE gels, and the separated protein bands were transferred electrophoretically to a polyvinylidene fluoride (PVDF) membrane. Immunoreactivity was determined based on enhanced chemiluminescence, and the signals were detected using a Bio-Rad ChemiDoc system (Bio-Rad Laboratories, China).

The following antibodies were used: anti-GAPDH (1:5,000, BM1623, Boster); anti-GluA1 (1:1,000, A1826, ABclonal); anti-mGluR1 (1:1,000, abx112750, Abbexa); anti-mGluR5 (1:1,000, A3758, ABclonal); anti-GluN2B (1:1,000, abx23583, Abbexa); anti-PSD-95 (1:1,000, abx236850, Abbexa); anti-α5 GABA (1:1,000, ab259880, Abcam). The secondary antibodies used were goat anti-rabbit IgG (1:10,000; Boster) and goat anti-mouse IgG (1: 10,000, Boster). The immunosignals were quantified using densitometry and were expressed relative to the GAPDH signals and normalized to the control for data analysis.

### Electron microscopy

The ultrastructures of the synapses in the mPFC, CeA, and hippocampal CA1 regions of rats from the sham, unsusceptible, and susceptible groups were examined using a transmission electron microscope (TEM) (Figure 1B). In brief, the respective regions were split and fixed successively in 2.5% glutaraldehyde (cold) and 1% OsO_4_ at 4°C (Mannaioni et al., 2001). After three washes with PBS, the specimens were dehydrated in a series of ethanol solutions. Subsequently, the samples were infiltrated and embedded in pure LR-White resin. Ten ultrathin sections (50 nm) were prepared from each region and then stained with uranyl acetate and lead citrate. The stained sections were observed under a JEM-1230 transmission electron microscope operating at 120 kV. Asymmetric (glutamatergic) synapses were characterized by thick electron-dense post-synaptic specializations while symmetric (GABAergic) synapses had thin post-synaptic specializations (Fitzgerald et al., 2019). Only the excitatory (asymmetric) synapses were examined.

### Statistical analysis

All result data were expressed as mean ± SEM and subjected to the analysis of significant differences between the mean values using a Tukey’s test after the normality distribution assessment that was evaluated using the Kolmogorov-Smirnov test. Hierarchical cluster analysis was performed according to a previous study conducted by our research group (Li et al., 2021). The rats that underwent the SMIR surgery were divided into the susceptible group (cognitively compromised pain rats) and the unsusceptible group (cognitively normal pain rats) based on the Ward method and Euclidean distance square as distance measurement using the hierarchical cluster analysis of the data from the NOP and Y-maze tests. The significance was tested using two-tailed tests, and a *p*-value of less than 0.05 was considered statistically significant. All graphs and statistical analyses were conducted using GraphPad Prism 8.0 and Adobe Illustrator Artwork 14.0.

## Results

### Skin/muscle incision and retraction-evoked significant mechanical hypersensitivity and lower cognition in rats

According to the results of a previous study, the SMIR-evoked mechanical hypersensitivity appeared by the postoperative Day 3, with the maximum level appearing between the postoperative Day 10 and Day 13, and persisted until the postoperative Day 22 (Flatters, 2008). Therefore, in the present study, the PWT was assessed on Day 8 or Day 9, and the cognitive behavior was evaluated on Day 9 or Day 10 after surgery (Figures 1A,B). According to our hierarchical clustering analysis of the cognitive behavior evaluation data (Figure 1C) from the NOP and Y-maze tests, the rats who underwent the SMIR surgery were divided into the susceptible group (*n* = 11) and the unsusceptible group (*n* = 5). As depicted in Figure 1D, the rats who underwent the SMIR surgery exhibited significantly decreased PWT. In the cognitive behavior test, the three groups exhibited no significant differences in terms of the total distance traveled (Figure 1E), suggesting no damage to motor function upon the SMIR surgery. In comparison to the sham and unsusceptible group rats, the rats in the susceptible group presented fewer entries into the new arm and exhibited a discrimination ability between the novel and the familiar object (Figures 1F,G), indicating poorer cognitive performance. Meanwhile, the sham group and the unsusceptible group rats exhibited no significant difference in this regard. These results verified that SMIR surgery leads to significant mechanical hypersensitivity and cognitive deficits in rats.

### Neuronal excitability alterations in the medial prefrontal cortex, central amygdala, and hippocampus pyramidal neurons after the skin/muscle incision and retraction surgery

**FIGURE 2:**
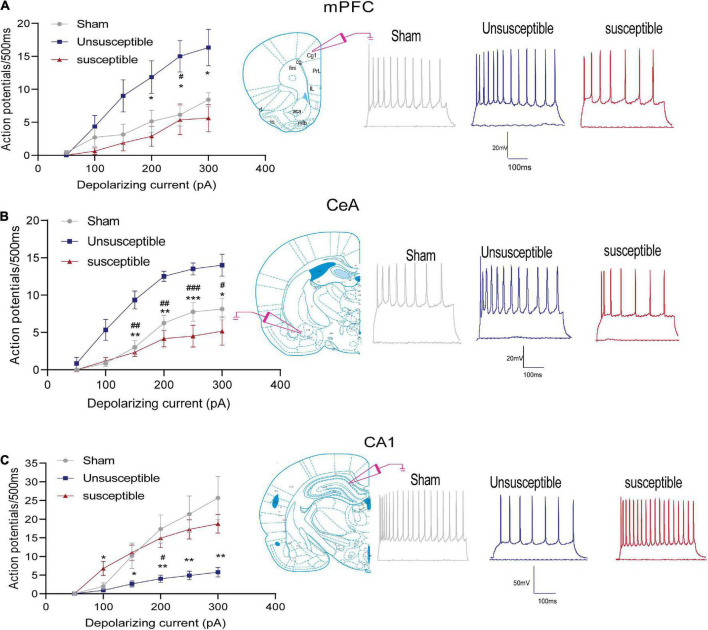
Neuronal excitability in the mPFC, hippocampus, and CeA pyramidal neurons after the SMIR surgery. Graphs present the mean frequency of AP (action potential) fired in response to the 500 ms current injection ranging from 0 to 300 pA (left). Traces present the potential firings evoked by the 500 ms depolarizing current steps of 300 pA (right). **(A)** Quantification of the AP firing frequencies in the anterior cingulate cortex of the mPFC neurons; *N* = 5 rats/group, *F* = 30.64, *p* < 0.0001. **(B)** Quantification of the AP firing frequencies in the CeA neurons; *N* = 5 rats/group, *F* = 18.16, *p* < 0.0001. **(C)** Quantification of the AP firing frequencies in the hippocampal CA1 neurons; *N* = 5 rats/group, *F* = 8.27, *p* = 0.0026. Statistical significance was determined by two-way ANOVA followed by Bonferroni’s *post-hoc* test. Sham vs. Unsusceptible, ^#^*p* < 0.05, ^##^*p* < 0.01, ^###^*p* < 0.001; Unsusceptible vs. Susceptible, *p < 0.05, **p < 0.01, ***p < 0.001.

The spiking frequency in the anterior cingulate cortex (the dorsal component of mPFC), CeA, and hippocampal CA1 excitatory neurons was determined to assess the intrinsic neuronal excitability after the SMIR surgery in all three groups (Figure 2). The number of depolarization-induced spikes generated upon intracellular injections of depolarizing current pulses was significantly different among the three groups. As depicted in the figure, the number of spikes in the mPFC and CeA neurons after SMIR was higher in the unsusceptible group rats compared to the sham group (Figures 2A,B), while the number of spikes in the susceptible group rats was lower than that in the unsusceptible group rats. On the contrary, neuronal excitability in the hippocampal CA1 neurons was decreased in the unsusceptible group rats and not in the susceptible group rats (Figure 2C). These results demonstrated pronounced differences in the intrinsic neuronal excitability in mPFC, CeA, and hippocampus pyramidal neurons between the CPSP rats with and without cognitive deficits.

### There were apparent discrepancies in the synaptic transmissions from the medial prefrontal cortex and central amygdala pyramidal neurons between the chronic postsurgical pain rats with and without cognitive deficits

**FIGURE 3:**
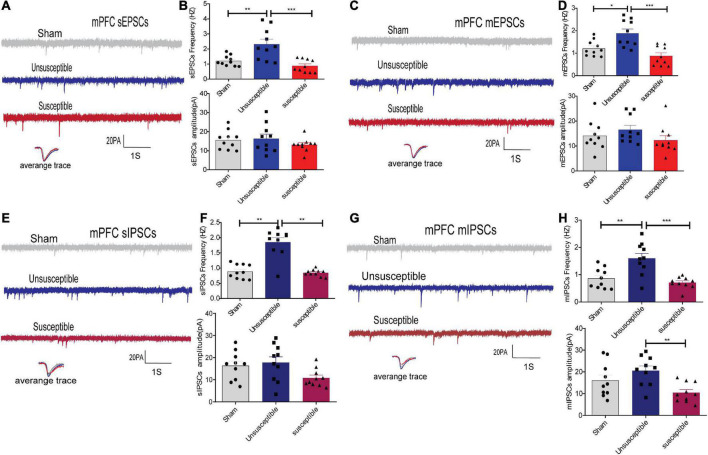
Apparent discrepancies in the synaptic transmissions in the anterior cingulate cortex of mPFC pyramidal neurons between the CPSP rats with and without cognitive deficits. **(A,C)** A typical time-course with traces of sEPSCs/mEPSCs in the individual slices from three groups of rats (above) and the individual traces (average) of sEPSCs/mEPSCs obtained from the corresponding recordings (bottom). Calibration: 20 pA. **(B,D)** Bar graphs presenting the frequencies and amplitudes of the sEPSCs/mEPSCs. sEPSCs (frequency: one-way ANOVA, *F* = 12.29, *P* = 0.0002; amplitude: one-way ANOVA, *F* = 0.94, *P* = 0.40), mEPSCs (frequency: one-way ANOVA, *F* = 11.98, *P* = 0.0002; amplitude: one-way ANOVA, *F* = 1.38, *P* = 0. 27). **(E,G)** Representative sIPSCs/mIPSCs in the pyramidal neurons recorded from the three groups of rats (above) and the individual traces (average) of sIPSCs/mIPSCs obtained from the corresponding recordings (bottom). Calibration: 20 pA. **(F,H)** Bar graphs presenting the frequencies and amplitudes of sIPSCs/mIPSCs. sIPSCs (frequency: Kruskal-Wallis test, *P* = 0.0009; amplitude: one-way ANOVA, *F* = 3.21, *P* = 0.06), mIPSCs (frequency: one-way ANOVA, *F* = 12.86, *P* = 0.0001; amplitude: one-way ANOVA, *F* = 6.16, *P* = 0.006). Results are expressed as mean ± SEM; *n* = 10 neurons from 5 rats/group; Tukey’s *post-hoc* tests; *p < 0.05, **p < 0.01, ***p < 0.001 (compared to sham and unsusceptible groups, respectively).

**FIGURE 4:**
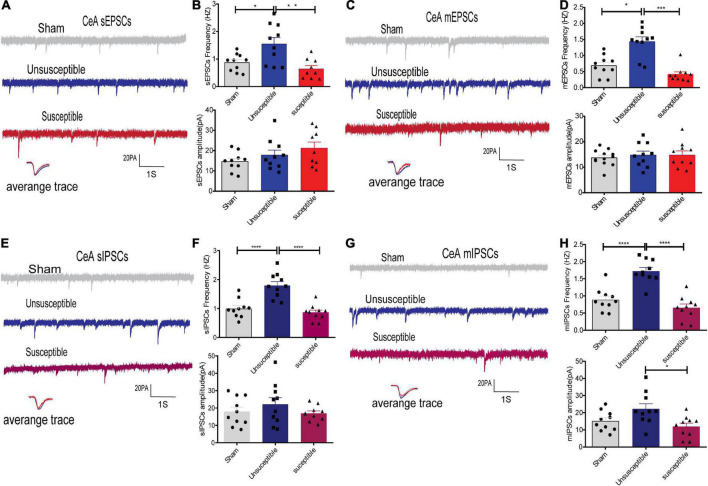
Evident differences in the synaptic transmission from the CeA pyramidal neurons between the cognitively normal and cognitively compromised pain rats. **(A,C)** Sample recordings of sEPSCs/mEPSCs in the individual slices from the three groups (above) and the individual traces (average) of sEPSCs/mEPSCs obtained from the corresponding recordings (bottom). Calibration: 20 pA. **(B,D)** Bar graphs presenting the frequencies and amplitudes of sEPSCs/mEPSCs. sEPSCs (frequency: one-way ANOVA, *F* = 8.46, *P* = 0.002; amplitude: one-way ANOVA, *F* = 2.02, *P* = 0.15), mEPSCs (frequency: Kruskal-Wallis test, *P* = 0.0002; amplitude: one-way ANOVA, *F* = 0.24, *P* = 0. 79). **(E,G)** Representative sIPSCs/mIPSCs in the pyramidal neurons from the three groups (above) and the individual traces (average) of sIPSCs/mIPSCs obtained from the corresponding recordings (bottom). Calibration: 20 pA. **(F,H)** Bar graphs presenting the frequencies and amplitudes of the sIPSCs/mIPSCs. sIPSCs (frequency: one-way ANOVA, *F* = 21.48, *P* < 0.0001; amplitude: one-way ANOVA, *F* = 0.98, *P* = 0.39), mIPSCs (frequency: one-way ANOVA, *F* = 28.23, *P* < 0.0001; amplitude: one-way ANOVA, *F* = 5.13, *P* = 0.01). Results are expressed as mean ± SEM; *n* = 10 neurons from 5 rats/group; Tukey’s *post-hoc* tests; *p < 0.05, **p < 0.01, ***p < 0.001, ****p < 0.001 (compared to sham and unsusceptible groups, respectively).

In order to evaluate whether alterations in synaptic transmission participate in the development of the cognitive deficits induced by CPSP, the excitatory synaptic transmission in the anterior cingulate cortex (the dorsal component of mPFC) and CeA pyramidal neurons in brain slices were recorded. The electrophysiology data suggested that compared to the sham group, the unsusceptible group exhibited significantly enhanced frequency of sEPSCs/mEPSCs in mPFC (Figures 3A–D) and CeA (Figures 4A–D) excitatory neurons. Interestingly, compared to the unsusceptible group, the susceptible group exhibited decreased synaptic transmission. No noticeable difference could be observed in the amplitudes of the excited sEPSCs/mEPSCs.

Moreover, since Gamma-aminobutyric acid (GABA) systems play crucial roles in learning and memory (He et al., 2019), GABA-driven inhibitory synaptic transmission was also recorded in the present study. Similarly, the frequency of sIPSCs/mIPSCs in the mPFC and CeA neurons exhibited the same tendency as that of the sEPSCs/mEPSCs from the three groups. As depicted in Figures 3E–H, 4E–H, the frequency of sIPSCs/mIPSCs in the mPFC and CeA neurons from the unsusceptible group was elevated. In addition, the amplitude of the mIPSCs of the mPFC and CeA neurons in susceptible group rats was lower than that in the unsusceptible group rats. These results suggested discrepancies in both excitatory and inhibitory synaptic transmission between the CPSP rats with and without cognitive deficits in the mPFC and CeA neurons.

### Synaptic transmissions in the hippocampal CA1 pyramidal neurons varied markedly between the chronic postsurgical pain rats with and without cognitive deficits

**FIGURE 5:**
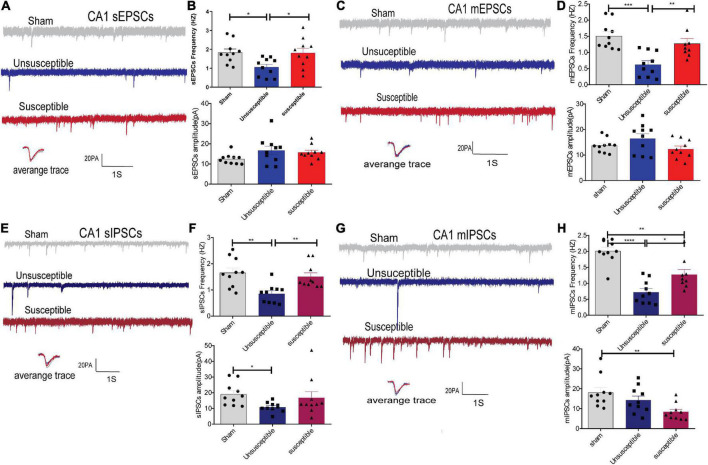
CPSP rats with and without cognitive deficits exhibited marked differences in the synaptic transmissions in hippocampal CA1 pyramidal neurons. **(A,C)** A typical time-course with traces showing a continuous recording of sEPSCs/mEPSCs taken from groups after SMIR surgery. Calibration: 20 pA. **(B,D)** Bar graphs present the frequencies and amplitude of sEPSCs/mEPSCs. sEPSCs (frequency: one-way ANOVA, *F* = 4.94, *P* = 0.01; amplitude: one-way ANOVA, *F* = 2.31, *P* = 0.12), mEPSCs (frequency: one-way ANOVA, *F* = 11.81, *P* = 0.0002; amplitude: one-way ANOVA, *F* = 0.41, *P* = 0. 11). **(E,G)** Representative sIPSCs/mIPSCs recorded in the CA1 pyramidal neurons from the three groups (above) and the individual traces (average) of sIPSCs/mIPSCs obtained from the corresponding recordings (bottom). Calibration: 20 pA. **(F,H)** Bar graphs presented the frequencies and amplitudes of sIPSCs/mIPSCs. sIPSCs (frequency: Kruskal-Wallis test, *P* = 0.0002; amplitude: Kruskal-Wallis test, *P* = 0.02), mIPSCs (frequency: one-way ANOVA, *F* = 26.28, *P* < 0.0001; amplitude: Kruskal-Wallis test, *P* = 0.004). Results are expressed as mean ± SEM; *n* = 10 cells from 5 rats/group; Tukey’s *post-hoc* tests; *p < 0.05, **p < 0.01, ***p < 0.001, ****p < 0.001 (compared to sham and unsusceptible groups, respectively).

Since the hippocampus plays a significant role in pain and cognition, the synaptic transmissions in the hippocampal CA1 neurons of the rats from the three groups were also recorded in the present study. As depicted in Figure 5, compared to the sham group, the unsusceptible rats exhibited a significant decline in the frequencies of sEPSCs/mEPSCs and sIPSCs/mIPSCs in the hippocampal CA1 neurons. Meanwhile, compared to the unsusceptible group, the susceptible group exhibited a significant elevation in the frequencies of sEPSCs/mEPSCs and sIPSCs/mIPSCs in the hippocampal CA1 neurons. Moreover, the amplitudes of the sIPSCs in the unsusceptible group and those of mIPSCs in the susceptible group were lower than those in the sham group (Figures 5F,H). These results revealed that the excitatory and inhibitory synaptic transmissions in the CA1 neurons differed markedly between the CPSP rats with or without cognitive deficits.

### Changes in the protein levels of mGluR1, mGluR5, GluN2B, GluA1, PSD-95, and α5-gamma-aminobutyric acid in medial prefrontal cortex, central amygdala, and hippocampus after the skin/muscle incision and retraction surgery

Considering the discrepancies in the synaptic transmissions from the mPFC, hippocampus, and CeA regions of the mouse brain among the three groups, the levels of synaptic transmission-related proteins were evaluated next. GluA1, which is one of the subunits of AMPAR (a-amino-3-hydroxy-5-methyl-4-isoxazole propionic acid receptor), and NMDAR mediate most of the excitatory synaptic transmissions, while GABAR mediates most of the inhibitory synaptic transmissions. A dysregulation in the levels of PSD95 and GABA reportedly contributes to pain and memory impairment (Perez-Sanchez et al., 2017; Coley and Gao, 2019; Kumari et al., 2020), while reduced PSD95 levels are predictive of cognitive deficits (Whitfield et al., 2014). In view of the role of Group I mGluRs (mGluR1 and mGluR5) in pain (Zhou et al., 2017) and cognition (Liu et al., 2012), the protein levels of mGluR1 and mGluR5 were also determined in the present study.

**FIGURE 6:**
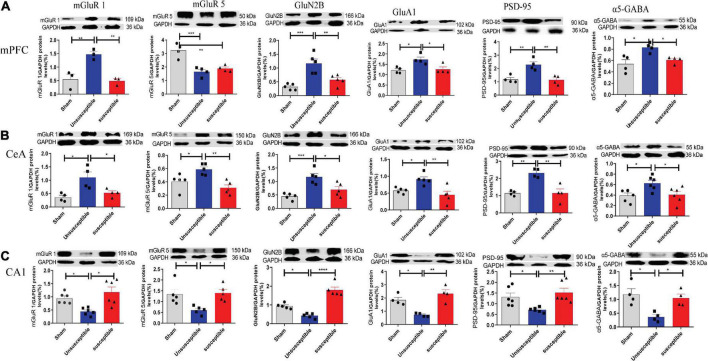
Changes in the levels of mGluR1, mGluR5, GluN2B, GluA1, PSD-95, and α5-GABA in mPFC, CeA, and hippocampus, 11 days after the SMIR surgery. **(A)** The representative Western blots and quantification of synaptic transmission-related proteins in mPFC. one-way ANOVA, mGluR1 (*F* = 16.24, *P* = 0.004), mGluR5 (*F* = 19.42, *P* = 0.0005), GluN2B (*F* = 16.28, *P* = 0.0004), GluA1 (*F* = 7.54, *P* = 0.02), PSD-95 (*F* = 13.36, *P* = 0.002), and α5-GABA (*F* = 8.31, *P* = 0.009). **(B)** Changes in the levels of synaptic transmission-related proteins in CeA. one-way ANOVA, mGluR1 (*F* = 8.23, *P* = 0.009), mGluR5 (*F* = 9.72, *P* = 0.003), GluN2B (*F* = 13.58, *P* = 0.0008), GluA1 (*F* = 9.05, *P* = 0.004), PSD-95 (*F* = 16.19, *P* = 0.001), and α5-GABA (*F* = 5.74, *P* = 0.01). **(C)** Changes in the expression levels of synaptic transmission-related proteins in the hippocampus. one-way ANOVA, mGluR1 (*F* = 9.03, *P* = 0.002), mGluR5 (*F* = 6.85, *P* = 0.01), GluN2B (*F* = 44.96, *P* < 0.0001), GluA1 (*F* = 15.50, *P* = 0.001), PSD-95 (*F* = 7.17, *P* = 0.006), and α5-GABA (*F* = 8.26, *P* = 0.009). Results are expressed as mean ± SEM; Tukey’s *post-hoc* tests; *p < 0.05, **p < 0.01, ***p < 0.001, ****p < 0.0001; *n* = 3–6/group.

The western blotting results revealed a significant increase in the post-SMIR protein levels of mGluR1, GluN2B, GluA1, PSD-95, and α5GABA in mPFC and CeA in the unsusceptible group compared to the sham group (Figures 6A,B), which was not observed in the susceptible group. Meanwhile, the levels of mGluR5 in mPFC in the unsusceptible group and susceptible group were lower than those in the sham group. Similarly, in the unsusceptible group, the protein levels of mGluR1, mGluR5, GluN2B, GluA1, PSD-95, and α5GABA from the hippocampus were decreased, while the same was not true for the susceptible group (Figure 6C). Collectively, these results indicated that the levels of synaptic neurotransmitter receptors differed significantly between the CPSP rats with cognitive deficits and the CPSP rats without cognitive deficits.

### The ultrastructures of the synapses from the medial prefrontal cortex, hippocampal CA1, and central amygdala regions varied in their postsynaptic density after the skin/muscle incision and retraction surgery

**FIGURE 7:**
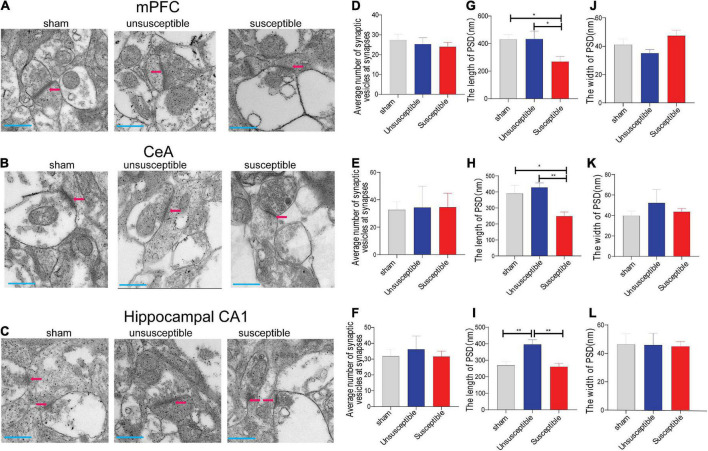
Electron microscopic images for the mPFC, CeA, and hippocampus regions after the SMIR surgery. **(A–C)** Representative synapses of the synapse ultrastructure in the mPFC, CeA, and hippocampus neurons. **(D–F)** The average number of synaptic vesicles at the synapses (*n* = 5 slices). one-way ANOVA, mPFC (*F* = 0.31, *P* = 0.73), CeA (*F* = 0.01, *P* = 0.99), and hippocampus (*F* = 0.18, *P* = 0.83). **(G–I)** The length of PSD (*n* = 5 slices). one-way ANOVA, mPFC (*F* = 4.54, *P* = 0.02), CeA (*F* = 6.30, *P* = 0.01), and hippocampus (*F* = 10.35, *P* = 0.0005). **(J–L)** The width of postsynaptic density (PSD) (*n* = 5 slices). one-way ANOVA, mPFC (*F* = 2.97, *P* = 0.07), CeA (*F* = 0.42, *P* = 0.66), and hippocampus (*F* = 0.01, *P* = 0.99). Scale bar = 500 nm. Results are expressed as mean ± SEM; Tukey’s *post-hoc* tests; *p < 0.05, **p < 0.01.

In order to establish that the excitatory and inhibitory synaptic transmission disorders were due to the presynaptic or postsynaptic deficits, the synaptic vesicles and postsynaptic density (PSD) in the anterior cingulate cortex (the dorsal component of mPFC), hippocampus, and CeA specimens from the three groups were investigated (Figures 7A–C). The electron microscopy results presented in Figures 7D–F revealed no significant difference in the number of synapses among the groups at the presynaptic level. However, at the postsynaptic level (Figures 7G,H), the length of PSD in the mPFC and CeA specimens from the susceptible group rats was lower than the corresponding value in the sham and unsusceptible group rats. In addition, the length of PSD in the hippocampus of rats from the susceptible group was reduced compared to the unsusceptible rats (Figure 7I). However, compared to the sham group, the unsusceptible group exhibited an elevated length of PSD in the hippocampus region of rats after SMIR (Figure 7I). No noticeable difference could be observed in the width of PSD in the three groups (Figures 7J–L). Collectively, these results suggested significant discrepancies in the synapse ultrastructure that were closely related to CPSP and the associated cognitive dysfunction in rats.

## Discussion

Substantial evidence suggests the detrimental effect of chronic pain on cognition (Cowen et al., 2018; Matthewson et al., 2019; Phelps et al., 2021). Nonetheless, whether cognitively compromised animals with pain have the exact same synaptic mechanisms as those in the cognitively normal animals with pain remains to be elucidated so far. The present study revealed that nearly two-thirds of the CPSP rats suffered cognitive impairment, which is consistent with the findings of previous clinical studies (Dick and Rashiq, 2007; Berryman et al., 2013). The most exciting finding of the present study was that spontaneous synaptic transmissions differed significantly between the CPSP rats with and without lower cognition. This finding was further corroborated by the variations observed in the levels of synaptic transmission-related proteins and the synapse ultrastructure. Overall, using a combination of different approaches, it was identified that CPSP rats with lower cognition exhibited strikingly different synaptic transmission compared to the CPSP rats without lower cognition. This finding would further advance the current understanding of the relationship between pain and pain-related cognition.

The proteins mGluR1 and mGluR5 are implicated in pain and cognition. These proteins play diverse roles in increasing neuronal excitability by modulating various ion channels and the associated proteins in the mPFC, CeA, and hippocampal CA1 pyramidal neurons (Mannaioni et al., 2001). For instance, in CA1 pyramidal cells, mGluR1 activation reportedly led to somatic calcium transients and direct neuronal depolarization, while mGluR5 activation inhibited the gradual after-hyperpolarization potential potassium currents and enhanced the NMDA receptor currents (Niswender and Conn, 2010). The accumulation of the GluN2B-NMDA receptors at PSD was anticipated to intensify a range of Ca^2+^–sensitive signaling cascades, which would contribute to the increased neuronal excitability and enhanced responsiveness to peripheral stimuli (Qiu et al., 2011). The present study revealed that neuronal excitability was increased in the mPFC and CeA neurons in cognitively normal rats with pain. This was consistent with the previous studies that reported increased excitability in the mPFC pyramidal cells after peripheral inflammation (Ong et al., 2019) and in the CeA pyramidal neurons in acute arthritis and neuropathic pain models (Jiang et al., 2014; Thompson and Neugebauer, 2019). Accordingly, a significant increase in the levels of mGluR1, mGluR5, and NMDAR-subunit N2B proteins was observed in the CeA of the cognitively normal pain rats. Furthermore, the neuronal excitability in the hippocampal CA1 neurons of cognitively normal pain animals was decreased. These results were consistent with the previous reports that reported prolonged decreases in the cellular activity of CA1 pyramidal neurons in response to painful stimuli (McEwen, 2001) and the association of the increased neuronal excitability in the CA1 pyramidal neurons with cognition function (Wang W. et al., 2020). As expected, the levels of mGluR1, mGluR5, and NMDAR-subunit N2B proteins in hippocampal CA1 were significantly reduced in the cognitively normal pain animals.

So far, it was clear that the alterations in the neuronal excitability due to dynamic changes in the levels of mGluR1, mGluR5, and NMDAR-subunit N2B proteins in the mPFC, CeA, and hippocampal CA1 pyramidal neurons were involved in the development of CPSP and the associated lower cognition. However, the causal relationship between neuronal excitability and mechanical hypersensitivity and the lower cognition of animals remained unclear. It is noteworthy that additional evidence concurs the deactivation of mPFC during chronic pain (Wang et al., 2015). These discrepancies could be explained by the differences observed in the record regain, animal strains, pain models, and points in time. In addition, Ji et al. (2010) reported that modulation of CeA does not affect arthritis pain-related dysfunction of decision-making. Again, this discrepancy could be partially explained by the difference in the animal models used or the different aspects of cognitive function.

PSD-95 is a major scaffolding protein in the PSD of excitatory synapses. PSD-95 plays a critical role in pain and cognition-related glutamatergic synaptic transmission by interacting with and functionally influencing the AMPA (α-amino-3-hydroxy-5-methyl-4-isoxazole propionic acid) and NMDA receptors (Qiu et al., 2011; Ong et al., 2019). For instance, PSD-95 deficiency is reported to disrupt the synaptic NMDAR/AMPAR current and the levels of associated proteins in the hippocampus and mPFC, thereby inducing learning and working memory deficits (Chen et al., 2015; Coley and Gao, 2019). In addition, an increase in the PSD-95 protein levels reflects an enhanced number of synapses (Seo et al., 2014), while aberrant regulation of GluA1 functions or dynamics is directly associated with cognitive impairment (Wang et al., 2017). Therefore, reduced PSD-95 or GluA1 levels are routinely used for predicting cognitive deficits (Whitfield et al., 2014; Ding et al., 2016). Interestingly, in the present study, the PSD-95 or GluA1 protein levels in the hippocampus were lower than in the cognitively normal pain animals compared to cognitively compromised pain animals, which was consistent with previous reports (Nyffeler et al., 2007; Leuba et al., 2008; Uchimoto et al., 2014). According to previous reports, an increase in PSD-95 expression could suggest compensatory phenomena related to *de novo* PSD-95 synthesis (Nyffeler et al., 2007; Leuba et al., 2008), while an increased GluA1 level could reduce the synaptic capacity for additional GluA1 trafficking that is associated closely with cognitive function (Uchimoto et al., 2014). Moreover, these explanations were consistent with the conclusion that cognitive ability could not be enhanced simply by increasing synaptic GluA1 accumulation and, rather, the newly added receptors should possess normal properties in the dynamics (Wang et al., 2017).

The inhibitory neurotransmitter GABA is a potent regulator of pain, learning, and memory via an ongoing level of tonic inhibition mediated primarily by extra-synaptic α5GABA_A_ receptors (Wang et al., 2012; Bravo-Hernandez et al., 2016). It has been consistently demonstrated previously that GABA concentrations are positively correlated with painful stimulus (Hasanein et al., 2008; Kupers et al., 2009). Conversely, a negative correlation exists between GABA concentrations and cognitive failure (Duncan et al., 2014; Prevot et al., 2019; Jimenez-Balado and Eich, 2021). However, the present study revealed that the level of the hippocampus α5GABA_A_ receptor was reduced in cognitively normal pain animals, which could be explained by the fact that α5GABA_A_ receptors also promote nociception levels by modulating the loss of GABAergic inhibition (El-Hassar et al., 2007). In addition, it was revealed that α5GABA_A_ levels in the hippocampus were higher in the cognitively compromised pain animals compared to the cognitively normal pain animals. This was in agreement with the previous reports stating that increasing the GABA_A_ receptor activity remarkably impeded memory (Wang et al., 2012; Whissell et al., 2013). This could be partially due to the attenuated network excitability and synaptic plasticity resulting from enhanced GABA_A_ activity constraining the neuronal firing in the hippocampal CA1 region.

Evidence suggests that a solid operative GABAergic inhibition could dampen an increased excitation via G-protein-activated inward rectifying potassium channels (El-Hassar et al., 2007; Berry-Kravis et al., 2018). Conversely, the activation of mGluR5 receptors upon glutamate release attenuates the release of GABAergic vesicles via CB1 receptors (Ji and Neugebauer, 2011). Furthermore, multiple data demonstrate that pain and the associated cognitive deficits are complex disorders related to receptor metabolism and the functional balance between glutamate and GABA neurotransmission (Duncan et al., 2014; Zhang et al., 2019). As expected, the present study also revealed an unbalanced excitatory/inhibitory ratio in synaptic transmission. For instance, in mPFC and CeA, the alterations in the inhibitory synaptic transmission included both presynaptic and postsynaptic mechanisms, while the alterations in the excitatory synaptic transmission mainly involved the presynaptic mechanism in the rats with and without poor cognition. It should be mentioned that the changed properties of hippocampus CA1 are significantly opposite to mPFC and CeA. A possible explanation for this diversity might be due in part to the diverse effects of mGluR5 in rodent hippocampus vs. mPFC and CeA. As mGluR5 are primarily postsynaptic in hippocampus neurons to weaken synaptic functions, but are both pre- and post-synaptic in mPFC and CeA neurons known to normalize sensory gating deficit, thus improving cognitive function (Yang et al., 2021).

Spontaneous glutamatergic/GABAergic synaptic transmission is assessed based on sEPSCs/sIPSCs, while mEPSCs/mIPSCs reflect the spontaneous single synaptic response to presynaptic individual synaptic vesicles or quanta (Kaeser and Regehr, 2014; Li et al., 2016). The frequency of spontaneous synaptic transmission is often interpreted as a representation of the presynaptic release probability and the number of available synaptic vesicles, while the amplitude depends on both presynaptic released-vesicle size and the number of postsynaptic receptors (Hung et al., 2014; He et al., 2019). The present study revealed apparent discrepancies in the frequencies and amplitudes, particularly in the former, of the synaptic transmission between the CPSP rats with and without cognitive deficits. The additional data on presynaptic vesicles and PSD corroborated these discrepancies.

At the level of presynaptic status, the available data indicated that the number of synaptic vesicles could not provide adequate evidence regarding the changes in the synaptic transmission frequency linked with chronic pain and the associated cognitive deficits. As the number of synaptic vesicles in the mPFC, CeA, and hippocampal CA1 appeared to remain unaltered, the observed alterations in the frequencies of sEPSCs/sIPSCs and mEPSCs/mIPSCs were most probably due to different probabilities of glutamate or GABA quanta release from the presynaptic terminals (Hung et al., 2014; He et al., 2019). Whether the release probability of synaptic vesicles in the active zones differs distinctly among the three groups evaluated in the present study warrants further detailed investigation.

At the level of postsynaptic status, the changes in the levels of postsynaptic receptors could precisely account for the discrepancies in the amplitudes of synaptic transmission related to pain-associated cognitive deficits. Moreover, PSD in the cognitively compromised pain animals had reduced length, which confirmed that PSD was positively correlated with cognitive function (Coley and Gao, 2019). It is noteworthy that the PSD length in the hippocampal CA1 was increased in cognitively normal pain animals. Since the enhanced synaptic information communication induced by the pain stimulus would require a greater area to operate, it was supposed that the longer length of PSD was a response to the painful stimulus. Therefore, it is suggested that the release probability of presynaptic vesicles, postsynaptic receptors, and PSD could be involved in the changes and discrepancies in synaptic transmission linked to the chronic pain and the associated cognitive deficits in rats.

Five key points from the present study are noteworthy and should be addressed in future studies. First, the abundant evidence in favor of synaptic structure and function in neural circuits resulting in behavioral consequences, including pain and impaired cognition (Li et al., 2018), is not adequate to prove whether the molecular and synaptic changes in the neurons were the reasons for the pain and the associated cognitive deficits. Second, LTP, a lasting enhancement of synaptic transmission efficacy, is considered the foundation for learning and memory. Previous studies have reported comprehensive data on the synaptic plasticity mechanism of pain and cognition (Kodama et al., 2007; Xiong et al., 2020; Zhang et al., 2020; Phelps et al., 2021). Therefore, initially, the present study was also aimed to determine the spontaneous synaptic transmission and the related molecular level distinction between pain and the associated cognitive deficits. Third, a potential target for ameliorating pain and the associated cognitive deficits must be clarified further. Fourth, throughout the present study, it was apparent that no differences existed between cognitively compromised pain animals and sham controls. The literature has been consistent on a negative correlation between synaptic transmission and cognitive impairment in various models (Wong et al., 2006; Schofield et al., 2011; Ding et al., 2018; Tantra et al., 2018), while a positive correlation is reported between synaptic transmission and pain (Blom et al., 2014; Hung et al., 2014; Cordeiro Matos et al., 2015; Wang Y. J. et al., 2020). Since the cognitively compromised pain animals are with cognitive impairments, it was postulated that the alteration in the synaptic transmission related to cognitive impairments could be offset by the synaptic transmission related to chronic pain. However, this hypothesis warrants further investigation for validation. Another explanation may be that the observed changes are specific for some compensatory mechanisms involved in the cognitively normal CPSP rats. Fifth, whether female rats would exhibit the same tendency as the male rats remained to be explored.

In conclusion, the present study is a pioneer in demonstrating that both synaptic structure and function were significantly different between cognitively compromised pain rats and cognitively normal pain rats and that this distinction was mainly due to presynaptic and postsynaptic modulation. Moreover, these findings strongly indicated that chronic pain and the associated cognitive functions could not be ameliorated simply by increasing or decreasing synaptic transmission-related receptor accumulation. Hence, although it remains unclear as to how the observed differences in these brain regions reveal any relationships between CPSP and cognitive performance, any further attempt at exploring chronic pain and the associated cognitive impairment should consider these discrepancies in the synaptic structure and function.

## Data availability statement

The original contributions presented in this study are included in the article/supplementary material, further inquiries can be directed to the corresponding author/s.

## Ethics statement

The animal study was reviewed and approved by the Animal Care and Use Committee, Tongji Hospital.

## Author contributions

ZL and HX designed the experiments and wrote the manuscript. ZL, ZH, ZXL, TS, and WZ performed the experiments. ZL and ZH analyzed the data. All authors revised and approved the final version of the article.
